# The importance of developmental assets in HIV prevention behaviors among young black men who have sex with men (MSM)

**DOI:** 10.1038/s41598-024-63123-7

**Published:** 2024-05-28

**Authors:** Donte T. Boyd, Orlando O. Harris, Gamji Rabiu Abu-Ba’are, LaRon Nelson, Leo Wilton

**Affiliations:** 1 College of Social Work, The Ohio State University, 1047 College RD, #325K, Columbus, OH 43215 USA; 2 Center for Interdisciplinary Research on AIDS (CIRA), Yale University, School of Public Health, New Haven, CT USA; 3 Center for AIDS Prevention Studies (CAPS), Division of Prevention Science, Department of Medicine, University of California, San Francisco, San Francisco, CA USA; 4 Department of Community Health Systems, School of Nursing, University of California, San Francisco, CA USA; 5 Behavioral, Sexual and Global Health Lab, School of Nursing,, University of Rochester, Rochester, NY USA; 6 Yale School of Nursing, Yale University, New Haven, CT USA; 7 Department of Human Development, Faculty of Humanities, State University of New York at Binghamton, Binghamton, NY USA; 8 Faculty of Humanities, University of Johannesburg, Johannesburg, South Africa

**Keywords:** HIV infections, Diseases

## Abstract

Developmental assets are critical to the health and wellbeing of youth. The current study examines the influence of developmental assets on PrEP use and HIV testing among YBMSM ages 18–24. Using a cross-sectional survey of YBMSM (N = 225), this study explored the role of external (e.g., family support, other adult support) and internal (e.g., personal responsibility) assets in explaining HIV prevention behaviors. Participants were recruited from Mechanical Turk (M-Turk) internet-based platform, social media sites, and community-based organizations. A path analysis was conducted to investigate the direct/indirect effects of internal and external assets on PrEP use and HIV testing. Family support (β = 0.40, *p* < 0.001) and other adult support (β = 0.22, *p* = 0*.*004) were both associated with personal responsibility. Personal Responsibility (β = 0.15, *p* = 0.03) and positive identity (β = 0.28, *p* < 0.001) were both associated with an increase HIV testing. Personal responsibility was positively associated with increased PrEP use (β = 0.30, *p* < 0.001). Our study results indicated that external assets play a role in helping to build internal assets that support increased HIV testing and PrEP use among YBMSM. Our findings suggest the need for strength-based interventions that help YBMSM build assets and increase HIV prevention behaviors.

## Introduction

Young Black men who have sex with men (YBMSM) continue to face the highest HIV infection rates in the United States^1,2^, with a 50% chance of HIV acquisition in their lifetime, which possibly reaches 60% by age 40^3^. Thus, the Centers for Disease Control and Prevention (CDC) recommends annual or more frequent screening for those demonstrating a higher risk of HIV infection for this population^4,5^. Despite the availability of HIV PrEP (pre-exposure prophylaxis), uptake has remained low among YBMSM since 2012^5,6^. HIV testing is the critical gateway to receipt of PrEP and rapid treatment among HIV negative persons for whom prophylaxis is indicated^5,7–10^. The Developmental Assets Framework lens offers a potential approach for preventing new HIV infections in this population.

### Developmental assets framework

The Developmental Assets Framework (DAF) is a comprehensive approach to understanding youth and the resources required for positive youth development^11,12^. It integrates evidence associated with lower-risk behaviors and promotes well-being^11–15^, impacting ecological and individual factors for YBMSM and other youth. The Search Institute (a research organization that is renowned for developing the developmental assets framework) identifies 40 youth developmental assets with 20 external (e.g., community, family support, social support, and neighborhood environment) and 20 internal assets (e.g., commitment to learning, positive values, social competencies, and positive identity)^11–15^. More assets correspond to fewer risk behaviors and increased positive outcomes. Consideration of the intersecting identities of YBMSM is crucial for understanding how assets function in different contexts based on the significant benefits associated with developmental assets. Previous research has shown that having more assets is linked to reduced levels of suicide ideation, violence, and depression^12,13^. In a recent study involving a national sample of YBMSM aged 18–29 (N = 400), researchers examined external assets like family support and open communication in relation to positive attitudes toward PrEP and PrEP stigma. The findings indicated that family support decreased PrEP stigma, while discussing sex and drugs with parents increased it. This study was the first to apply the DAF to HIV prevention behaviors in YBMSM^16^. However, additional research is necessary to gain a deeper understanding of how the DAF can positively impact HIV-related outcomes among YBMSM. This framework holds promise for designing and implementing HIV prevention programs aimed at increasing HIV testing and PrEP use within this population. Guided by DAF, this study seeks to investigate whether external assets (e.g., family support) and internal assets (e.g., personal responsibility) impact HIV testing and PrEP use among YBMSM**.**

### External and internal assets

Studies investigating the DAF have focused on external resources like family support and communication^11^. Notably, youth well-being is significantly influenced by support from family, other adults, friends, and romantic partners, particularly for LGBTQ + youth^17–19^. Findings from these studies suggest that supportive families increase the likelihood of disclosure of sexual orientation^12^. Communication between parents and adolescents has also been found to be essential for healthy development, to reduce sexual risk behaviors, and to promote safer sexual practices^20–25^. While studies find that parents could be the primary source of sex education^26^, many avoid discussing sex with their children^27^. Limited research, however, has explored family communication among YBMSM regarding HIV prevention, and due to challenges like limited sexual health communication within families, YBMSM often seek support from other community adults. Therefore, further research is needed to involve parents and supportive adults in HIV prevention to support PrEP uptake among YBMSM. Internal assets are the personal skills, commitments, and values that can help YBMSM make healthier choices, take responsibility for their own lives, and be independent and fulfilled^10,11^. Prior research suggests that family support is linked to self-esteem and positive identity and has been shown to reduce depression and suicidality^10,11^. Another study reported that internal assets (e.g., personal responsibility) were a predictor of life satisfaction among Latino youth^28^. Other research indicates self-identity disclosure is associated with positive outcomes^29^. However, limited research has explored internal assets for LGBTQ + youth^15^ including YBMSM. This limits our understanding of the impact of internal assets on the health behaviors of YBMSM; meanwhile, cultivating assets at a young age can have positive lifelong implications.

### Study purpose

The objective of this study, incorporating a developmental assets framework^16^, is to investigate how external and internal assets influence HIV prevention behaviors among YBMSM. For this study, we tested a total of 15 assets, including both internal and external ones.

**Figure 1:**
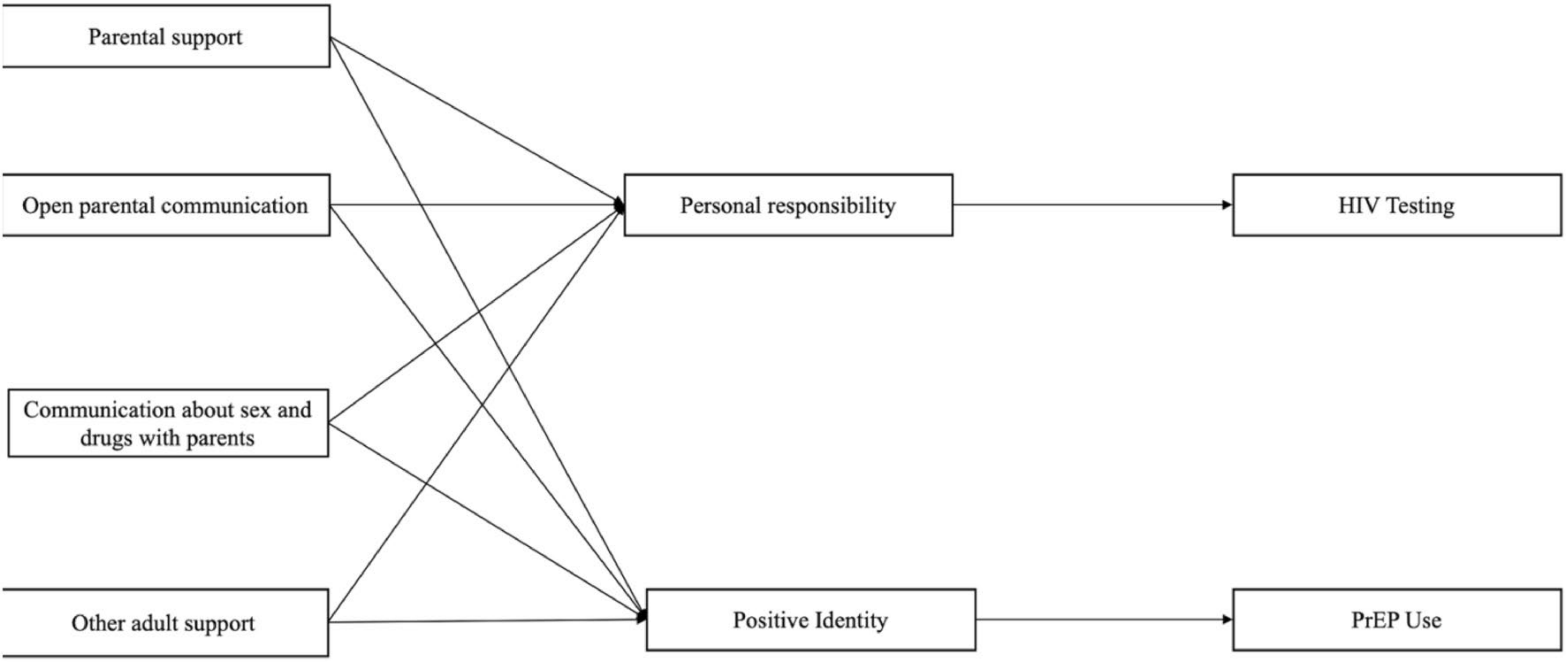
Developmental assets and HIV prevention model.

This is the one of the first studies to examine internal assets as mediators for YBMSM. Our first hypothesis posits that external assets are associated with internal assets. Secondly, we hypothesize that internal assets will be associated with increased HIV testing and PrEP use. Our third hypothesis proposes that internal and external assets indirectly affect HIV prevention efforts, HIV testing, and PrEP utilization (See Fig. 1).

## Methods

### Ethical statement

This cross sectional study was conducted in compliance with the guiding principles of the Declaration of Helsinki and received approval from The Ohio State University Institutional Review Board (IRB #2021E1175). Prior to the study, all subjects provided written informed consent.

### Study procedures and recruitment

This study used data from a larger study that examined strength-based approaches in relation to the sexual, physical, and mental health, as well as suicidal behaviors, of young Black men who have sex with men aged 18–29. The data was stratified to investigate how assets impact HIV prevention outcomes among individuals specifically aged 18–24 (n = 225). The survey was programmed with Qualtrics software for different sampling sites. An anonymous link was generated and included on a recruitment flyer, which was then distributed via social media sites (Facebook and Twitter) and provided to community-based organizations and Amazon Mechanical Turk (M-Turk)^16,30^. The principal investigator and research assistants distributed the survey via social media every morning at 8 a.m. Eastern Time.

Amazon M-Turk offers cost-effective and rapid recruitment for research across various fields, including public health^31^. To access and take part in the survey, M-Turk registrants were required to have a HIT approval rating of 95% or higher from previous surveys, be at least 18 years old, and live in the United States, as confirmed during their initial M-Turk registration. HIT approvals are essential for evaluating and endorsing tasks completed by participants on the platform, with the Approval Rate representing the percentage of HITs approved by participants for surveys they have completed^16,30^. Furthermore, individuals logging into the M-Turk platform during the survey week were informed of the opportunity to take a survey focused on HIV and assets for Black MSM. They were told that the survey would require approximately 20 min and would be available daily at 8 a.m. Eastern Standard Time. Participants were instructed to complete the survey in one session and receiving a one-dollar compensation and other incentives (e.g., gift cards) from M-Turk^16,30,31^.

The research team collaborated with community-based organizations, providing them with the survey flyer. Community health workers, in turn, shared this flyer with their eligible clients, including an anonymous survey link. Recruitment occurred between December 1, 2021, and January 31, 2022. Participants who completed the 20-min survey and provided their email received a $35 Amazon gift card. For data quality and to prevent bots, our survey employed Qualtrics survey protection. We also verified respondents’ IP addresses to confirm their residence in the United States, thus ensuring data integrity by preventing duplicate responses to eligibility and survey questions. Furthermore, we used speeding checks to exclude participants with survey durations less than one-third of the median survey duration from the final sample. Qualtrics survey protection provided tools to prevent fraudulent submissions, including a ballot box stuffing prevention tool that places a browser cookie after a response, reCAPTCHA scores, which required respondents to identify items in pictures or replicate letters, and bot detection through a reCAPTCHA score indicating the likelihood of a respondent being a bot.

### Participants

Consistent inclusion/exclusion criteria were applied across all sampling sites. Eligible participants for the study were those who self-identified as Black or African American, were aged 18–24, lived in the United States, were assigned male at birth, were proficient in English, currently identified as a man, and reported sexual contact with a male in the past year. Respondents who did not meet these criteria were promptly exited from the survey. We enforced a forced response option in Qualtrics to ensure every participant answered each question. After clicking the survey link, participants received information about the study and were requested to complete a screening tool to determine their eligibility. The verified participants answered questions on demographics, developmental assets, and HIV prevention behaviors.

Majority of the sample identified as Black or African American (94%), followed by Caribbean (4%), Afro-Latino (1%) and 1% self-identified as continental African. Thirty one percent of the sample never attended high school, while 33% completed college or were post graduate. The average household income ranged from ˂ $20,000 to $150,000, the average being $57,499. All the participants (100%) self-reported having anal sex with another male within the last 12 months; 54% reported being gay, 27% straight or heterosexual, 16% bisexual, 1% questioning, and 2% other.

### Measures

#### Outcome variable

PrEP use was measured using a single item based on a dichotomous response (0 = No, I have not used PrEP in the past 12 months, and 1 = Yes, I have used PrEP in the past 12 months). Participants were asked the following question and given the following definition: “Have you used PrEP in the past 12 months? PrEP is medicine people at risk for HIV take to prevent getting HIV from sex or injection drug use.”

*HIV testing* was defined by participants responses to the following: “Have you, yourself, ever been tested for HIV in the last 12 months?” The responses were coded using a dichotomous response (0 = “No”, 1 = “Yes”).

#### Mediators

##### Internal assets

*Positive Identity* was measured using six items based on a 5-point Likert scale ranging from 1 (*Strongly Disagree*) to 5 (*Strongly Agree*). *Positive Identity* asset category was recognized into two assets: hope (a combination of personal power, sense of purpose, and positive view the future) and self-esteem (three items; subset of items from Rosenberg). Participants were asked questions such as “I feel I do not have much to be proud of”. The Cronbach alpha was 0.90^32^. *Personal Responsibility* was measured using three items based on a 5-point, Likert scale ranging from 1 (*Strongly Disagree*) to 5 (*Strongly Agree*). Participants were asked statements such as “Telling the truth, even when it’s not easy”. The Cronbach’s alpha was 0.88^32^.

#### Independent variables

##### External assets

*Parental Support* was measured using three items based on a 5-point, Likert scale ranging from 1 (*Strongly Disagree*) to 5 (*Strongly Agree*). Participants were asked statements such as “My parents give me help and support when I need it.” We averaged responses to these three items, with higher scores indicating more parent support^16,33^. The Cronbach’s alpha was 0.95. *Open parent communication* was measured using a single item with values ranging from 1 (*Strongly Disagree*) to 5 (*Strongly Agree*). Participants were asked to rate the following statement: “I have lots of good conversations with my parents”^16,32^. Higher scores indicated increased open family communication. *Communication with parents about sex and drugs* was measured using a single item, rated from 1 (*Never*) to 5 (*All Of The Time*), which asked respondents, “If you had an important concern about drugs, alcohol, sex, or some other serious issue, would you talk to your parent(s) about it?”^16,33^. Higher scores indicated an increase in communication with parents about sex and drugs. *Other Adults Support* was measured using a single item, with values ranging from 1 (*Strongly Disagree*) to 5 (*Strongly Agree*), asking participants to rate the statement, "Adults in my town or city make me feel important.” A higher score indicated that adults in the community support the young men^16,32^.

### Statistical analysis

**Figure 2:**
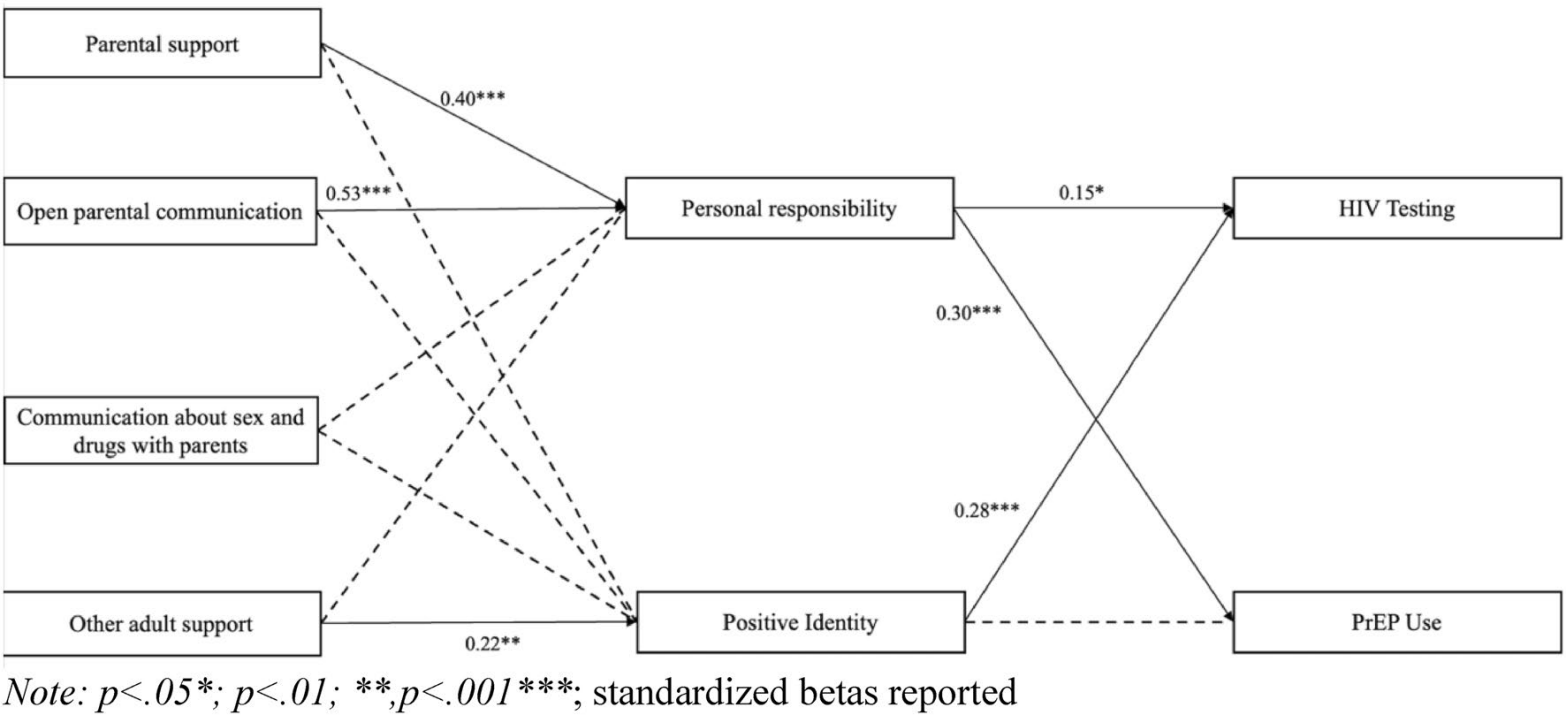
Path model testing direct and indirect effects from external to PrEP use and HIV testing through external assets (n = 225).

Table 1 provides means and standard deviations for the study’s continuous variables. Table 2 provides frequencies and percentages of categorical variables. M-plus version 8.7 was used to conduct a path analysis to test hypotheses examining whether external and internal assets were associated with HIV testing and PrEP use (Table 3, Fig. 2). Path analysis also allowed us to test the indirect effects of external assets on HIV test and PrEP use (Table 4). We applied the means- and variance-adjusted weighted least squares estimator instead of maximum likelihood estimation, as this estimator is preferred when the dependent variable is categorical and when the data are not normally distributed^33^. The percentage of missing data was ˂ 5%. Regarding missing data, we used full information maximum likelihood^33^. The goodness-of-fit was assessed with measures using the chi-square test, Akaike information criterion (AIC) and Bayes information criterion (BIC) because the dependent variable was categorical. Alternatively, we included correlation coefficients and standardized beta coefficients and *p*-values to examine the associations among the study variables.

**Table 1:** Means, standard deviations, and ranges of continuous variables (*N* = 225).

Variable	Mean	Standard deviation	Range
Age	22.0	1.92	18–24
Parent support	3.78	1.01	1.0–5.0
Open parent communication	3.84	1.07	1.0–5.0
Communication with parents about sex and drugs	3.41	1.22	1.0–5.0
Other adult support	3.77	1.00	1.0–5.0
Personal responsibility	4.01	0.75	1.0–5.0
Positive identity	3.34	0.78	1.0–5.0

**Table 2:** Frequency and percentages of categorical study variables (*N* = 225).

Variable	Frequency	Percentage
Mother education		
Less than high school	11	5
Some high school	13	6
High school diploma and GED	75	33
Some college or Associates degree	33	15
College degree or post graduate	73	32
I don’t know	20	9
Father education		
Less than high school	15	6
Some high school	20	9
High school diploma and GED	42	19
Some college or Associates degree	30	13
College degree or post graduate	30	13
I don’t know	88	40
Housing situation (who do you live with)		
Living with parents	100	44
Sibilings	50	22
Grandparents	7	3
Other family members	11	4
Friends	17	7
Parnter	24	11
Roommates	11	4
Other	5	2
Disclosed of sexuality		
Parents		
Yes	130	58
No	95	42
Use of preexposure prophylaxis		
Yes	178	79
No	47	19
HIV testing		
Yes	190	84
No	35	16

**Table 3:** Direct of effects on internal assets, HIV testing and PrEP use through external assets (N = 225).

Coefficients	B	β	SE	95% CI
Developmental assets				
Personal responsibility				
Parental support → personal responsibility	0.30***	0.40***	0.08	0.14,0.47
Open parent communication → personal responsibility	0.01	− 0.02	0.08	− 0.17,0.14
Communication about sex and drugs with parents → personal responsibility	0.08	− 0.12	0.05	− 0.18,0.03
Other adult support → personal responsibility	0.17**	0.22**	0.06	0.05,0.28
Positive identity				
Parental support → positive identity	0.42***	0.53***	0.09	0.25,0.59
Open parent communication → positive identity	− 0.06	− 0.09	0.08	− 0.22,0.10
Communucation about sex and drugs with parents → positive identity	− 0.10	− 0.16	0.06	− 0.21,00
Other adult support → Positive identity	0.10	− 0.12	0.06	− 0.03,0.21
HIV prevention behaviors				
HIV testing				
Personal responsibility → HIV testing	0.08*	0.15*	0.04	0.01,0.16
Positive identity → HIV testing	0.15***	0.28***	0.04	0.08,0.22
PrEP use				
Personal responsiblity → PrEP use	0.16***	0.31***	0.04	0.08,0.22
Positive identity → PrEP use	0.03	0.05	0.03	− 0.04,0.09

p < 0.05*, p < 0.01**, p < 0.001***; *B* unstandardized betas, *β* standardized betas, *SE* standardized errors, *CI* confidence intervals.

**Table 4:** Indirect effects on HIV testing and PrEP use through external and internal assets (N = 225).

	B	SE	95%CI
HIV testing			
Parental support → personal responsibility → HIV testing	0.10***	0.02	0.04,0.13
Open parental communication → personal responsibility → HIV testing	− 0.01	0.01	− 0.04,0.02
Communication about sex and drugs with parents → personal reponsibility → HIV testing	− 0.02**	0.01	− 0.04,0.00
Other adult suppport → personal responsibility → HIV testing	0.03**	0.01	0.00,0.05
PrEP use			
Personal responsibility → positive identity → PrEP use	0.06**	0.02	0.02,0.10
Open parental communication → positive identity → PrEP use	− 0.00	0.01	− 0.03,0.02
Communication about sex and drugs with parents → positive identity → PrEP use	− 0.01	0.01	− 0.03,0.00
Other adult support → positive identity → PrEP use	0.03**	0.01	0.01,0.05

p < 0.05*, p < 0.01**, p < 0.001***; *B* unstandardized betas, *SE* standardized errors, *CI* confidence intervals.

## Results

YBMSM reported high levels of parent support (*M* = 3.78, *SD* = 1.10), open parent communication (*M* = 3.84, *SD* = 1.07), other adult support (*M* = 3.77, *SD* = 1.00) and personal responsibility (*M* = 4.01, *SD* = 0.75). Communication about sex and drugs with parents (*M* = 3.41, *SD* = 1.22) and positive identity (*M* = 3.34, *SD* = 0.78) were both moderate (Table 1). Majority of the sample lived with their parents (44%) and their siblings (22%). Fifty eight percent of the sample disclosed their sexuality to their parents. Thirty three percent of their mothers had a high school diploma, and 19% of their fathers. Seventy-nine percent of the sample reported using PrEP in the past 12 months and 84% reported receiving an HIV test in the past 12 months (see Table 2).

### Path analysis

Parental support (β = 0.40, *p* < 0.001; 95% CI 0.14, 0.47) and other adult support (β = 0.22, *p* = 0.004; 95% CI 0.05, 0.28) were both directly and positively associated with personal responsibility. Parental support was directly and positively associated with positive identity (β = 0.53, *p* < 0.001; 95% CI 0.25, 0.59). Open parent communication and communication about sex and drugs were not directly associated with personal responsibility or positive identity. Other adult support was also not directly associated with positive identity (see Table 3).

Personal responsibility (β = 0.15, *p* < 0.001; 95% CI 0.01, 0.16) and positive identity (β = 0.28; p < 0.001; 95% CI 0.08, 0.22) were both directly and positively associated with HIV testing. Personal responsibility was directly and positively associated with PrEP use (β = 0.31; p < 0.001; 95% CI 0.08, 0.22). Positive identity was not directly associated with PrEP use (see Table 3).

Our results indicated that parental support (*B* = 0.10, *p* < 0.001; 95% CI 0.04, 0.13) and other adult support (*B* = 0.03, *p* < 0.001; 95% CI 0.00, 0.05) were indirectly and positively associated with HIV testing. However, communication about sex and drugs with parents was indirectly and negatively associated with HIV testing (*B* = − 0.02, *p* < 0.01; 95% CI − 0.04, 0.00). Parental support was indirectly associated with PrEP use (*B* = 0.06, *p* < 0.01; 95% CI 0.02, 0.10). Lastly, other adult support was also indirectly associated with PrEP use (*B* = 0.03 *p* < 0.01; 95% CI 0.01, 0.05). Open parent communication was not indirectly associated with HIV testing and PrEP use and communication about sex and drugs with parents was not associated with PrEP use (see Table 4).

## Discussion

Although young Black MSM are disproportionately affected by HIV, advancements in HIV testing and biomedical tools such as PrEP have presented the opportunity to help end the epidemic sooner rather than later. In this sample, 79% self-reported using PrEP, and 84% reported receiving an HIV test. Based on our knowledge, this is the first study to investigate how developmental assets—both internal and external—influence HIV testing and PrEP uptake. Consistent with prior literature, our results indicated that external assets such as family and other supportive adults contributed to increasing HIV testing among YBMSM^18,19,21^. In addition, external assets were associated with positive internal assets^18,20^. Our results also indicated external assets indirectly increased PrEP use. This finding is critical because PrEP uptake remains low among YBMSM. Notably, our results indicated that internal assets (personal responsibility and positive identity) directly increased HIV testing and PrEP use. There is important because there is a dearth of literature that has examined internal assets among LGBTQ + individuals. Overall, our results suggest that by integrating developmental assets into ongoing HIV prevention programs and interventions, the DAF may work similarly for increasing HIV testing rates and PrEP use among YBMSM.

Our results confirmed the first hypothesis that external assets were associated with internal assets. With family support, more YBMSM felt personally responsible and had a positive identity. Having other adult support was also associated with YBMSM feeling personally responsible, which contributes to the literature about the impact of family and other adult support on responsibility among YBMSM. It is plausible that families and other positive adults have healthy boundaries and relationships with their sons and other young people in the community, or that families and some adults effectively communicate when engaging with young people^20^. A sense of personal responsibility and an understanding of what responsibility means may help the overall development and engagement of YBMSM in safer sex practices.

Similar to prior literature, our study linked family support to positive identity^12,13^. Parent and family support shapes the development of many young adults, and in this study sample, majority self-reported that they had positive family support, suggesting that there may be healthy family dynamics that allows YBMSM to be more comfortable in who they are as individuals. Family Support was also indirectly associated with HIV testing and PrEP use. This is consistent with prior literature and extends the literature to include PrEP use and YBMSM^20^. Prior longitudinal studies indicated that family support increased HIV testing among Black youth^20,21^. Results also indicated other adult support was indirectly associated with HIV testing and PrEP use. The support that these young men may feel within their families and from other adults may influence their decision-making around their sexual behaviors and whether or not they engage in HIV prevention behaviors such as HIV testing and PrEP use. These findings also suggest that having a village of positive support may be critical to the well-being of YBMSM. These findings suggest that external assets play a critical role in young Black males’ development and engagement in healthy behaviors, including HIV prevention behaviors. Thus, researchers and practitioners should consider ways to include external assets in culturally tailored interventions on HIV.

Findings from our study suggest that internal assets were associated with both HIV testing and PrEP use. These findings are consistent with those from prior literature, and show that the more assets youth have, the more likely they will engage in positive behaviors such as life satisfaction^12–15^. YBMSM may feel it is their personal responsibility to get tested for HIV and use PrEP to protect themselves and others. Positive identity was also associated with HIV testing among YBMSM. Most of the literature that has examined positive identity among Black LGBTQ people have examined it as a part of sexual orientation disclosure^12,13^; only few studies have examined its influence on HIV testing, and none among YBMSM^34^. This finding is consistent with prior literature that suggests positive identity influences HIV testing. However, this study extends how positive identity is operationalized by moving past mere disclosure of one’s sexual orientation to including personal power, sense of purpose, positive view of the future, and self-esteem. This is critical because a positive identity can influence the overall health and well-being of YBMSM. Future research is needed to understand how developmental assets influence HIV prevention behaviors and other health outcomes.

Although the present study contributes to the knowledge-base of developmental assets and their positive or protective effect, it is not exempt from limitations. This study did not use all 40 developmental assets, which means that it cannot state what other assets may increase HIV testing and PrEP use among YBMSM. This was a national sample of YBMSM; however, it was not a representative sample, and as such, the results may not be generalizable. Furthermore, this was a cross-sectional study; hence, causality cannot be determined. The temporal link between the outcome and the exposure cannot be determined because both are examined simultaneously.

Despite these limitations, the present study provides research on the direct and indirect effects between developmental assets and HIV prevention behaviors among YBMSM. Our findings suggest that developmental assets can help increase the low numbers of YBMSM engagement in PrEP use and HIV testing. In addition, findings suggest that interventions are needed to help young men and people cultivate external and internal assets.

YBMSM continue to be burdened by HIV despite the effectiveness of biomedical preventions like PrEP and HIV testing. However, the developmental assets framework offers a way to increase engagement in these prevention efforts. Developmental assets have a positive influence on a plethora of outcomes such depression, suicidality and now HIV testing and PrEP use. The present study adds to the HIV and positive youth development literature by bringing to the table critical findings regarding the associations between developmental assets, and HIV prevention behaviors for YBMSM. The developmental assets framework gives us an opportunity to humanize YBMSM in society and promote a positive attitude and wellbeing.

## Data Availability

The datasets generated and analysed during the current study available from the corresponding author on reasonable request.
